# Prediction of dose-dependent in vivo acetylcholinesterase inhibition by profenofos in rats and humans using physiologically based kinetic (PBK) modeling-facilitated reverse dosimetry

**DOI:** 10.1007/s00204-021-03004-4

**Published:** 2021-03-02

**Authors:** Isaac Omwenga, Shensheng Zhao, Laetitia Kanja, Hans Mol, Ivonne M. C. M. Rietjens, Jochem Louisse

**Affiliations:** 1 Division of Toxicology, Wageningen University and Research, Stippeneng 4, 6708 WE Wageningen, The Netherlands; 2 Department of Public Health, Pharmacology and Toxicology, Faculty of Veterinary Sciences, University of Nairobi, P.O. Box 29053-00625, Nairobi, Kenya; 3 Wageningen Food Safety Research, Wageningen University and Research, Akkermaalsbos 2, 6708 WB Wageningen, The Netherlands; 4 Department of Animal Science, Meru University of Science and Technology, P.O. Box 972-60200, Meru, Kenya

**Keywords:** Organophosphate pesticides (OPs), Physiologically based kinetic (PBK) modeling, Reverse dosimetry, Acetylcholinesterase (AChE) inhibition, Novel approach method (NAM)

## Abstract

Organophosphate pesticides (OPs) are known to inhibit acetylcholine esterase (AChE), a critical effect used to establish health-based guidance values. This study developed a combined in vitro–in silico approach to predict AChE inhibition by the OP profenofos in rats and humans. A physiologically based kinetic (PBK) model was developed for both species. Parameter values for profenofos conversion to 4-bromo-2-chlorophenol (BCP) were derived from in vitro incubations with liver microsomes, liver cytosol, and plasma from rats (catalytic efficiencies of 1.1, 2.8, and 0.19 ml/min/mg protein, respectively) and humans (catalytic efficiencies of 0.17, 0.79, and 0.063 ml/min/mg protein, respectively), whereas other chemical-related parameter values were derived using in silico calculations. The rat PBK model was evaluated against literature data on urinary excretion of conjugated BCP. Concentration-dependent inhibition of rat and human AChE was determined in vitro and these data were translated with the PBK models to predicted dose-dependent AChE inhibition in rats and humans in vivo. Comparing predicted dose-dependent AChE inhibition in rats to literature data on profenofos-induced AChE inhibition revealed an accurate prediction of in vivo effect levels. Comparison of rat predictions (BMDL10 of predicted dose–response data of 0.45 mg/kg bw) and human predictions (BMDL10 of predicted dose–response data of 0.01 mg/kg bw) suggests that humans are more sensitive than rats, being mainly due to differences in kinetics. Altogether, the results demonstrate that in vivo AChE inhibition upon acute exposure to profenofos was closely predicted in rats, indicating the potential of this novel approach method in chemical hazard assessment.

**Supplementary Information:**

The online version contains supplementary material available at 10.1007/s00204-021-03004-4.

## Introduction

Organophosphorus or organophosphate pesticides (OPs) have been extensively used for the control of agricultural and household pests globally (Kumari and John 2019). Exposure to these pesticides has been reported for various segments of the population, including agriculture workers and their families, house hold members during home application of pesticides, people that live in proximity to farms, or the general public via residues on food (Bradman et al. 2003; Lu et al. 2000; Quandt et al. 2004). Consequently, OPs and their metabolites have been found in human blood, serum, urine, and breast milk (Liu et al. 2014; Hardt and Angerer 2000; Zhang et al. 2014; Naksen et al. 2016). Since various OPs have shown to cause adverse effects to the environment and to human health, many OPs are currently not registered for use as pesticides, and some OPs have even been banned (Hertz-Picciotto et al. 2018).

Epidemiological studies have linked chronic OP exposure to reproductive disorders, developmental toxicity, birth defects, cancer, Parkinson’s disease, Alzheimer’s disease, diabetes, chronic respiratory disease, cardiovascular diseases, chronic nephropathies and amyotrophic lateral sclerosis (ALS) (Mostafalou and Abdollahi 2013). The critical effects of OPs in animal studies are related to neurotoxicity, which has been reported to be related to, amongst others, inhibition of acetylcholinesterase (AChE), neuropathy target esterase (NTE) (Costa 2018), acylpeptide hydrolase (APH) (Richards et al. 2000), fatty acid amide hydrolase (FAAH) (Quistad et al. 2001; Buntyn et al. 2017), muscarinic M2 receptors (Costa 2006), and a variety of lipases (Quistad et al. 2006). Inhibition of AChE is one of the mechanisms by which OPs cause acute neurotoxicity (Jamal et al. 2002; Farahat et al. 2003), characterized by decreased hydrolysis of acetylcholine in both central and peripheral cholinergic synapses, resulting initially in overstimulation of nicotinic and muscarinic receptors, followed by receptor down-regulation on post-synaptic membranes (Costa 2006). Acute or repeated exposure to OPs can lead to organophosphate ester-induced delayed polyneuropathy (OPIDP), a neurodegenerative disorder caused by inhibition and aging of at least 70% of the activity of the neuropathy target esterase (NTE) present in nerve tissues as well as other tissues (e.g., lymphocytes, testis) (Johnson and Glynn 1995, 2001; Costa 2018). In the hazard and risk assessment of OPs, in vivo animal studies on OP-induced inhibition of AChE have been used to derive a point of departure (POD) to set safe exposure levels, such as an Acute Reference Dose (ARfD). ARfDs for chlorpyrifos, acephate, methamidofos, omethoate and profenofos as reported by organizations as the European Food Safety Authority (EFSA), United States Environmental Protection Agency (US EPA), and the Joint FAO/WHO Meeting on Pesticide Residues (JMPR) have been derived from data on OP-induced AChE inhibition from animal studies (JMPR 2007; EPA 2006, 2011, 2016; EFSA 2019). Although these in vivo studies do not measure complex neurotoxicity endpoints, the information on OP-induced inhibition of AChE in vivo is considered an important piece of information in the hazard characterization. In these in vivo studies, AChE activity is usually measured in blood and sometimes also brain tissue after OP exposure. In the present study, we aimed to assess whether in vivo dose-dependent OP-induced AChE inhibition can be predicted by an animal-free approach. To inhibit AChE in the in vivo situation, the OP needs to reach its target (AChE) at sufficiently high concentrations. The in vivo potency of an OP to inhibit AChE is, thus, dependent on its intrinsic ability (potency) to inhibit AChE and the amount of OP that reaches that target. The potency of an OP to inhibit AChE can be determined using in vitro approaches. To estimate the amount of OP that reaches the target, so-called physiologically based kinetic (PBK) models can be used. A PBK model permits the simulation of the chemical’s in vivo kinetics (ADME) and can relate external exposure to internal exposure at the target sites. When having PBK models for different exposure routes and/or different species, these models can be used for exposure route and/or species extrapolations. These models can also be used as a tool to estimate exposure applying reverse dosimetry of biomonitoring data (e.g., chemical levels in blood or urine), as has been shown, for example, for chlorpyrifos in rats and humans (Timchalk et al. 2002), and chlorpyrifos in children (Rigas et al. 2001). Furthermore, these models can be used for reverse dosimetry of in vitro toxicity data, thereby translating in vitro effect concentrations to in vivo doses, enabling prediction of in vivo dose-dependent toxicity, as has been shown for the OP chlorpyrifos (Zhao et al. 2019). Such PBK modeling-facilitated reverse dosimetry of in vitro toxicity data is considered crucial in the transition to non-animal based novel approach methods (NAMs) for the safety assessment of chemicals (Louisse et al. 2017).

PBK models have been developed for various OPs including chlorpyrifos (Timchalk et al. 2002; Bouchard et al. 2005; Mosquin et al. 2009; Lu et al. 2010; Zhao et al. 2019), malathion (Bouchard et al. 2003, 2017; Bogen and Singhal 2017), parathion (Sultatos 1990) and diazinon (Poet et al. 2004). To date, no PBK model has been built for the OP profenofos, despite its widespread use in developing countries and reported cases of human accidental poisoning (Gotoh et al. 2001; Eddleston et al. 2009).

**Fig. 1:**
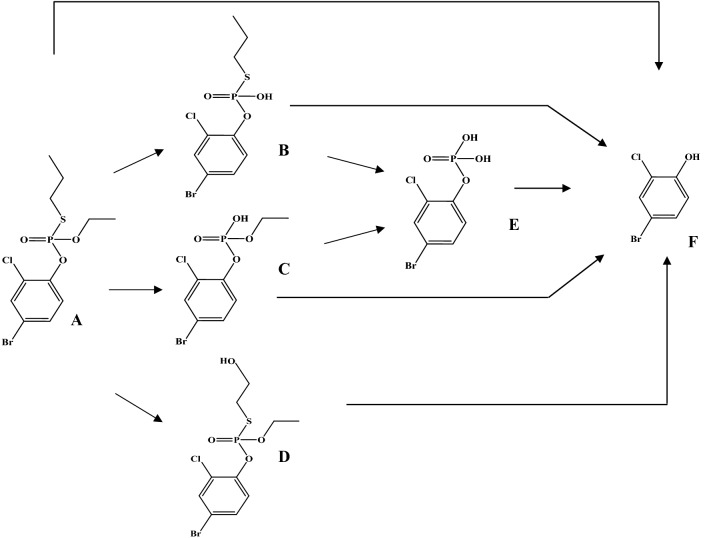
Metabolic pathways for metabolism of profenofos (**a**) based on data reported in previous studies (Gotoh et al. 2001; Dadson et al. 2013). Metabolites include desethylated profenofos (**b**), desthiopropylprofenofos/des-S-propylated profenofos (**c**), hydroxypropyl profenofos (**d**), 4-bromo-2-chlorophenyl dihydrogen phosphate (**e**), and 4-bromo-2-chlorophenol (BCP) (**f**)

Profenofos [*O*-(4-bromo-2-chlorophenyl) *O*-ethyl *S*-propyl phosphorothioate] (Fig. 1) is a thiophosphate OP pesticide (*O*=P–S–C) that was developed for pest strains resistant to chlorpyrifos and other OPs (Gotoh et al. 2001). Profenofos has been classified as a moderately hazardous (Toxicity Class II) pesticide by the World Health Organization (WHO) with moderate level of acute toxicity (LD50 of 358–1178 mg/kg in rat) following oral administration (Reported in JMPR (2007)). Dietary intake of profenofos is the primary exposure route for humans (Greish et al. 2011) and residue levels exceeding EU MRLs have been found in various vegetables in Kenya (Karanja et al. 2012). Our recent study reported profenofos as one of the most frequently encountered pesticide residues in vegetables sampled in peri-urban Nairobi, Kenya (Omwenga et al. 2021).

The present study aims to develop a PBK model for profenofos in rats and humans and apply the models to predict dose-dependent in vivo AChE inhibition using PBK modeling-facilitated reverse dosimetry of in vitro data on profenofos-induced AChE inhibition, allowing interspecies comparisons and providing a proof-of-principle that OP-induced AChE inhibition can be predicted for both rats and humans without the need for in vivo studies. To that end, profenofos PBK models were developed based on in silico- and in vitro-derived input parameter values. The rat PBK model was evaluated by comparing PBK model predictions with available in vivo kinetic data in rats. Subsequently, in vitro data on profenofos-induced AChE inhibition were translated to predicted in vivo dose-dependent AChE inhibition in rats and humans, and predictions on dose-dependent AChE inhibition in rats were evaluated by comparison with available in vivo data.

## Materials and methods

### Materials

Profenofos, 4-bromo-2-chlorophenol (BCP), bovine serum albumin (BSA), reduced nicotinamide adenine dinucleotide phosphate (NADPH), 5,5-dithiobis (2-nitrobenzoic acid) (DTNB) and acetylthiocholine iodide (ATC) were purchased from Sigma-Aldrich (Zwijndrecht, The Netherlands). Magnesium chloride hexahydrate (MgCl_2_⋅6H_2_O), trifluoroacetic acid (TFA), dimethylsulfoxide (DMSO), and calcium chloride dihydrate (CaCl_2_⋅2H_2_O) were purchased from VWR International (Amsterdam, The Netherlands). Acetonitrile (ACN, UPLC/MS grade) was purchased from Biosolve (Valkenswaard, The Netherlands). Human liver microsomes and cytosol (pooled from 150 human donors, mixed gender), rat liver microsomes and rat liver cytosol (pooled from 20 male Sprague–Dawley rats) were purchased from Corning (Amsterdam, The Netherlands). Recombinant human acetylcholinesterase was purchased from Sigma-Aldrich (Zwijndrecht, The Netherlands). Rat blood was purchased from BioIVT (Westbury, USA), human and rat plasma were purchased from Innovate (New York, USA).

### PBK model development

#### Model structure and software

**Fig. 2:**
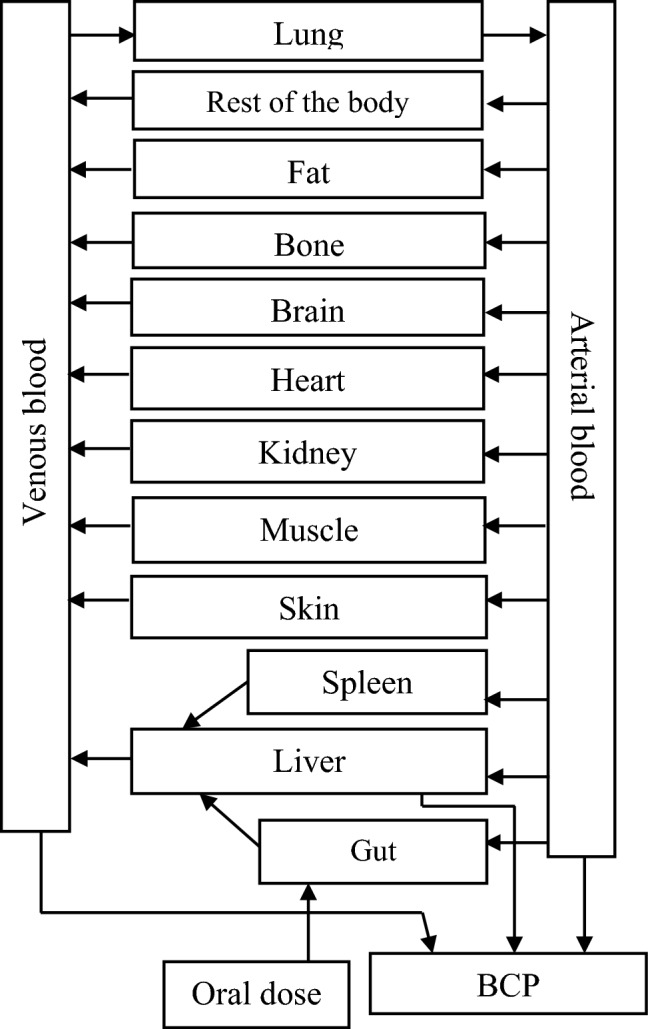
Schematic diagram of the PBK model for profenofos for rats and humans

The PBK model structure applied (Fig. 2) is based on a generic PBK model that has been developed for humans (Jones and Rowland-Yeo 2013). Physiological parameter values (tissue volumes and blood flows) for rats and humans were taken from Jones and Rowland-Yeo (2013) (Supplementary Table 1) as collected previously by Punt et al. (2020). The generic model contains separate compartments for liver, the gastrointestinal tract (GI-tract), fat, muscle, skin, bone, brain, heart, kidney, lung, spleen, venous blood, arterial blood, and a rest-of-body compartment. The uptake of profenofos from the GI-tract was described as a first-order process with an absorption rate constant of 1 h^−1^. All absorbed profenofos was assumed to be transferred directly to the liver compartment via the portal vein. Tissue:plasma partition coefficients were estimated based on the method of Berezhkovskiy (2004) using log*K*_*ow*_, pKa and fraction unbound plasma (fup) as input parameters. Log*K*_*ow*_ and pKa values were estimated using chemicalize (www.chemicalize.com) and fup was estimated (based on LogP and pKa) using the online simcyp tool (https://www.certara.com/software/pbpk-modeling-and-simulation/).

The main metabolite of profenofos reported in vivo is 4-bromo-2-chlorophenol (BCP), which has been reported to be formed upon paraoxonase 1 (PON1)-mediated hydrolysis of profenofos (JMPR 2007; EPA 2016; Dadson et al. 2013) (Fig. 1). Profenofos hydrolysis (BCP formation) was quantified in the present study using rat and human liver microsomes, liver cytosol and plasma (In vitro incubations to derive kinetic parameter values for description of metabolic clearance in the PBK model). Profenofos clearance was included in the blood and liver compartments of the PBK model. In in vitro studies, also CYP-mediated formation of other metabolites has been reported (in the presence of inhibitors of hydrolysis enzymes) (Dadson et al. 2013), but these are considered less relevant, since the hydrolysis reaction is more efficient than the CYP-mediated oxidations, as indicated in our in vitro metabolism studies with liver microsomes or cytosol in which practically all profenofos was converted into BCP. Since BCP was shown to not inhibit AChE (see "Results"), only profenofos kinetics were considered relevant for the reverse dosimetry and included in the PBK model (Fig. 2). The only in vivo data publicly available on profenofos kinetics in rats or humans, required for model evaluation, relate to the urinary excretion of conjugated BCP in rats. Therefore, to allow model evaluation, the rat PBK model was extended to include a sub-model on BCP and BCP-glucuronide formation (Supplementary Fig. 1). Estimated tissue:plasma partition coefficients of BCP and BCP-glucuronide and fraction unbound plasma (fup) values used for these sub-models are presented in Supplementary Table 1.

Renal excretion is also included in the model which is described by a passive renal clearance (via glomerular filtration) of the unbound fraction in blood at the rate of 6.7 L h^−1^ in humans and 0.078 L h^−1^ in rats. The model equations were coded and numerically integrated in Berkeley Madonna 9.1.18 (UC Berkeley, CA, USA), using the Rosenbrock’s algorithm for stiff systems. The PBK models’ differential equations are provided in the Supplementary Materials.

#### In vitro incubations to derive kinetic parameter values for description of metabolic clearance in the PBK model

Incubations of profenofos with rat and human liver microsomes, liver cytosol and plasma were performed to quantify in vitro rates of BCP formation. Conditions were optimized to be linear for metabolite formation with regard to incubation time and microsomal, cytosolic and plasma protein concentration (data not shown). The final incubations were carried out in 100 mM Tris HCl (pH 7.4, 37 °C) containing (final concentrations) 5 mM MgCl_2_, 2 mM CaCl_2_ [to stimulate PON1 activity (Carr et al. 2015)], 2 mM NADPH (cofactor to also include CYP-mediated metabolism), enzyme preparation (final concentration 2 mg/ml for human liver microsomes and cytosol and 4.4 mg/ml for human plasma; 0.5 mg/ml for rat microsomes and cytosol and 1.65 mg/ml for rat plasma) and profenofos (at final concentrations ranging from 1 to 100 μM, added from 100 times concentrated stock solutions in DMSO). Control incubations were performed in the absence of microsomes, cytosol or plasma. After 2 min pre-incubation, the reaction was initiated by adding the substrate and mixtures were incubated for 2 min in a 37 °C water bath. The total volume of the incubation mixtures was 200 μl. The reaction was terminated by the addition of 50 μl ice cold ACN and samples were kept on ice. The mixture was centrifuged at 14,000*g* for 20 min at 4 °C and the supernatant was analyzed using UPLC-UV.

#### UPLC-UV analysis

All samples from incubations were analyzed using a Waters Acquity UPLC H class system that consisted of a quaternary solvent manager, a sample manager, and a photodiode array (PDA) detector, equipped with a Water Acquity UPLC^®^ BEH C18 column (1.7 μm, 2.1 × 50 mm) and Waters Xbridge UPLC^®^ BEH C18 pre-column (2.5 μM, 2.1 × 5 mm). The temperature of the column was kept at 40 °C and the auto sampler at 10 °C during the UPLC analysis. The mobile phases used for the analysis consisted of (A) 0.1% TFA in nanopure water and (B) 100% ACN. A gradient elution at a flow rate of 0.6 ml/min was applied for the analysis with the initial condition of 100% A:0% B (v/v), changing in a linear way to 0% A:100% B from 0 to 6 min, which was maintained for 30 s, and then changed back to the initial conditions in 30 s, which were maintained for 1 min. The injection volume for each sample was 3.5 μl.

Under these conditions, the retention times of profenofos and BCP were 4.76 and 3.37 min, respectively. The amounts of profenofos and BCP were quantified by integrating the peak areas at 237 nm using calibration curves that were prepared using commercially available standards.

#### In vitro metabolism data analysis and scaling in the PBK model

Kinetic parameters including the apparent maximum velocity (Vmax) and the apparent Michaelis–Menten constant (Km) for BCP formation were obtained by fitting the data for the substrate concentration-dependent rate of conversion (expressed in nmol/min/mg protein) using GraphPad Prism 5, version 5.04 (San Diego, California, USA) to the standard Michaelis–Menten equation:

$$V\, = \,V_{{{\text{max}}}} *\left[ S \right]/({\text{Km}}\, + \,\left[ S \right])$$

in which the *S* represents the concentration of substrate, expressed in μM, *V* and *V*_max_ the velocity and the maximum velocity of the reaction, respectively, expressed in nmol/min/mg protein, and Km the apparent Michaelis–Menten constant, expressed in μM. The kinetic parameter values for conversion of profenofos to BCP in the liver microsomes, liver cytosol and plasma were determined. To determine the catalytic efficiency, *V*_max_ was divided by the Km.

The in vitro *V*_max_ values were scaled in the PBK model code (Supplementary Materials) using the following scaling factors for rats and humans: 35 mg microsomal protein/g liver, 80.7 mg cytosolic protein/g liver and 550 mg plasma/g blood (Medinsky et al. 1994; Cubitt et al. 2011). The apparent Vmax values obtained from enzymatic incubations expressed in nmol/min/mg protein were converted into μmol/h/kg liver and plasma in the PBK model code. The in vivo Km values were assumed to be equal to those obtained in vitro (taking into account the differences in free fraction in vitro vs in vivo).

#### Model evaluation

Since the only in vivo data available on profenofos kinetics for model evaluation were on the urinary excretion of conjugated BCP in rats, the rat PBK model was extended to include a sub-model on BCP and BCP-glucuronide (Supplementary Fig. 1). Estimated partition coefficients and fup values of BCP and BCP-glucuronide used for these sub-models are presented in Supplementary Table 1 and conversion of BCP to BCP-glucuronide was assumed to take place only in the liver having the same apparent Km and *V*_max_ values as reported before by Strikwold et al. (2013) for phenol glucuronidation. The PBK model-predicted cumulative urinary excretion of BCP-glucuronide were compared with reported in vivo data on conjugated BCP excretion in profenofos-exposed rats (Cho et al. 2002).

Furthermore, a sensitivity analysis was performed to estimate the impact of the chemical-specific parameters on the model output [maximum free (unbound) blood concentrations of profenofos in this study]. Normalized sensitivity coefficients (SCs) were calculated using the following equation:

$${\text{SC}}\, = \,\left( {C^{\prime} - C} \right) \, / \, \left( {P^{\prime} - P} \right) \, \times \, (P/C).$$

in which *P* represents the parameter value in the PBK model and *P*′ the parameter value with a 5% increase (Evans and Andersen 2000). Likewise, *C*′ represents the model output obtained with the 5% increase in *P*, while *C* is the model output using the initial model parameter value. The sensitivity analysis was conducted for oral exposure to predicted effect doses obtained with the reverse dosimetry analysis (BMDL_10_ values). BMDL_10_ values were selected since a BMDL_10_ value from a rat study on profenofos-induced AChE inhibition was recently used to derive the POD to obtain an acute reference dose (ARfD) for profenofos by the US EPA (EPA 2016).

### Determination of in vitro AChE inhibition by profenofos and quantitative in vitro to in vivo extrapolation (QIVIVE) using reverse dosimetry

To perform reverse dosimetry of in vitro AChE inhibition by profenofos, first the in vitro AChE activity in the absence or presence of increasing concentrations of profenofos was determined. For humans, commercially available recombinant AChE was used. For rats, no commercially available recombinant AChE was available, so rat AChE was obtained from rat blood as described below.

#### Derivation of a concentration–response curve for human recombinant AChE inhibition

AChE inhibition was determined following the protocol of Ellman et al. (1961) as modified by Chambers and Chambers (1989). The concentration of acetylthiocholine and recombinant AChE were optimized to be linear for metabolite formation (data not shown). Human recombinant AChE was dissolved in 0.1 M sodium phosphate (pH 7.4) containing 1 mg/ml BSA (which was present to stabilize human recombinant AChE (Rosenfeld and Sultatos 2006)) to reach an enzyme concentration of 200 U/ml. Subsequently, the AChE enzyme solution was further diluted with 0.1 M sodium phosphate (pH 7.4) containing 1 mg/ml BSA to a working concentration of 1 U/ml. A range of concentrated stock solutions of profenofos were prepared in ethanol and 50 times diluted by adding 5 µl of stock solution to 245 µl of 0.1 M sodium phosphate (pH 7.4) (containing 0.1 mg/ml BSA). For the negative control, 5 µl of ethanol without profenofos was added to 245 µl of 0.1 M sodium phosphate (pH 7.4) (containing 0.1 mg/ml BSA). Subsequently, 5 µl of these working solutions was added into wells of a 96-well plate already containing 44-µl sodium phosphate (pH 7.4) (containing 0.1 mg/ml BSA). To start the reaction (in absence or presence of a range of profenofos concentrations), 1-µl enzyme solution was added, giving a total volume of 50 µl per reaction with an AChE concentration of 0.02 U/ml and an ethanol concentration of 0.2%, a level that had no effect on the activity of the enzyme. After 15-min incubation at 37 °C, the reactions were stopped by adding 150 µl of a mixture of 5,5-dithiobis (2-nitrobenzoic acid) (DTNB) and acetylthiocholine (ATC) (final DTNB and ATC concentrations were 0.075 and 0.15 mM, respectively). Subsequently, the time-dependent increase in absorbance due to formation of chromophore (DTNB + thiocholine) was measured at 37 °C using a wavelength of 412 nm during a period of 10 min, using a spectrophotometer (SpectraMax, Molecular Devices, UK).

AChE activity was expressed as enzyme activity (percent of control). The concentration of profenofos that produced a 50% decrease in AChE activity (IC50) was determined from best‐fit plots of the mean (± SD) percentages of inhibition vs. the 10log logarithm of profenofos concentrations using GraphPad Prism 5, version 5.04 (San Diego, California, USA) equation:

$$Y = 100/(1 + 10^{{(X - {\text{LogIC}}50)}} ).$$

#### Derivation of a concentration–response curve for rat erythrocyte AChE inhibition

##### Blood sample processing and enzyme extraction

Rat blood samples were processed according to a method reported by Larsen et al. (2019) with a few modifications to isolate the extrinsic membrane bound AChE. Briefly, the blood sample (2 ml) was centrifuged at 3000*g* for 15 min to separate plasma and cells. Aliquots of 500 µl of the cells were resuspended in 4.5 ml lysis buffer (20 mM sodium phosphate; pH 7.4) and frozen at – 80 °C for 24 h. After that, the cell samples were thawed and centrifuged (4000*g*) for 15 min at 4 °C. The supernatant was poured off and the precipitate was suspended in 4.5-ml lysis buffer. This treatment was repeated another two times. The residue containing what is called erythrocyte ‘ghost’ membranes, was resuspended in 500-µl analysis buffer (100 mM sodium phosphate; pH 7.4), and stored at − 80 °C until analysis. Aliquots of erythrocyte ‘ghost’ membrane preparations were used for protein measurement using the Pierce™ BCA Protein Assay Kit (Thermofisher) with BSA as standard for quantification (Lowry et al. 1951), and for AChE activity assessment.

##### Rat red blood cell (RBC) AChE activity

The effect of profenofos on rat AChE activity was assessed as described above for human recombinant AChE, with a few modifications. Briefly, a typical reaction mixture (200 µl) for AChE activity contained 0.005 mg/ml erythrocyte ‘ghost’ membrane protein. The rest of the steps were as described above for measurement of human recombinant AChE activity.

#### QIVIVE of AChE inhibition data with PBK modeling-facilitated reverse dosimetry

In the present study, it was assumed that in vivo dose-dependent AChE inhibition (in blood) depends on the maximum concentration (*C*_max_) of profenofos reached in the blood. For adequate PBK modeling-facilitated reverse dosimetry, the active (unbound) concentration of a test chemical in the in vitro test system should be linked to the in vivo freely available chemical at the target site. This is important since it is assumed that it is the fraction unbound that causes the effect (AChE inhibition). In the in vitro incubations, a very low concentration of BSA (0.1 mg/mL) is present. Heilmair et al. (2008) reported that with such low BSA concentrations, the free concentration of chlorpyrifos oxon is not affected. Furthermore, Rosenfeld and Sultatos (2006) found no evidence of binding of paraoxon by BSA even at a higher concentration (1 mg/ml) during incubations. Based on these observations, no significant effect on the free fraction of profenofos in the in vitro AChE inhibition studies is expected, which corroborated with estimations on profenofos binding in the in vitro incubations with help of the online simcyp tool (https://www.certara.com/software/pbpk-modeling-and-simulation/). A description of the fraction unbound in blood is incorporated in the PBK model, hence, the predicted in vivo unbound *C*_max_ values in blood were related to the profenofos concentrations we applied in vitro.

By calculating with the PBK model, the external dose required to reach (as unbound *C*_max_) the concentrations applied in the in vitro test, each in vitro concentration was translated into an in vivo dose. In this way, the concentration–response curves for rat and human AChE inhibition were converted into in vivo dose–response curves for profenofos-induced AChE inhibition in rats and humans, respectively.

### Evaluation of predicted dose–response curves for rat and human AChE inhibition

To evaluate the performance of the PBK modeling-facilitated reverse dosimetry approach to predict in vivo AChE inhibition, the predicted dose–response curve for rat AChE inhibition upon exposure to profenofos was compared with available in vivo data on AChE inhibition in rats (JMPR 2007). Furthermore, the predicted dose–response data were used for BMD modeling, using EFSA PROAST version 69.0 (https://shiny-efsa.openanalytics.eu/app/bmd) using the model averaging approach to allow evaluation of the prediction by comparison of BMDL_10_ values obtained from the predicted dose–response data to points of departure derived by regulatory bodies (EPA 2016; JMPR 2007, EFSA 2019). To that end, the obtained BMDL_10_ values were compared with a reported BMDL_10_ value from a rat study on profenofos-induced RBC AChE inhibition, which was recently used to obtain an ARfD by the US EPA (EPA 2016), and a reported NOAEL value on profenofos-induced rat brain acetylcholinesterase inhibition, which has been used to obtain an ARfD by JMPR (2007).

## Results

### In vitro conversion of profenofos to BCP

The conversion of profenofos to BCP was measured in incubations with both rat and human liver microsomes, cytosol and plasma. UPLC analysis revealed that only one peak (BCP) appeared when analyzing the samples obtained from these incubations. In control incubations, small amounts (max 0.04% of the amount of profenofos added) of BCP were detected, indicating some spontaneous hydrolysis of profenofos in the aqueous environment, which has also been observed in previous studies (Aly and Badawy 1982). The BCP formation data for microsomes, cytosol and plasma were, therefore, corrected for BCP levels detected in control incubations.

**Fig. 3:**
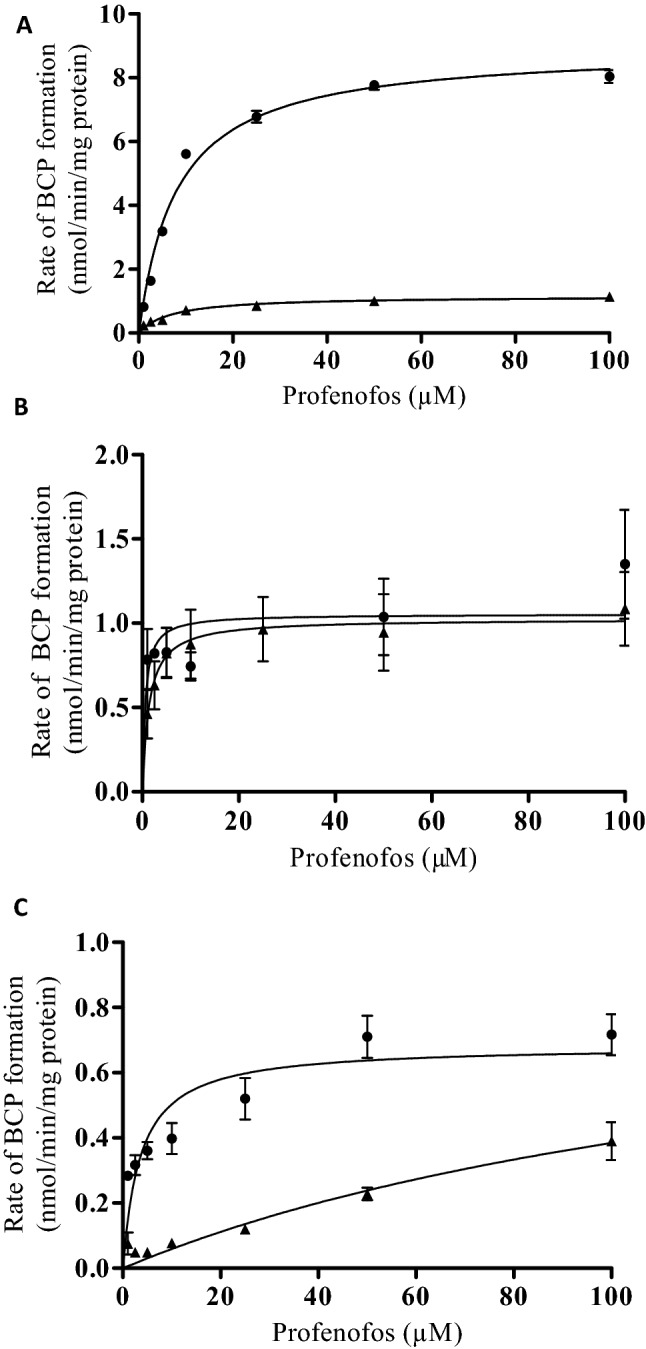
Concentration-dependent rate of profenofos conversion to BCP in incubations with human (triangles) and rat (dots) **a** liver microsomal proteins, **b** liver cytosolic proteins, and **c** plasma proteins. Results represent data from 3 independent experiments and are presented as mean ± SEM

The concentration-dependent velocity of BCP formation following incubation of profenofos with both human and rat liver microsomes, liver cytosol and plasma is depicted in Fig. 3a–c. The kinetic parameters derived from these data (Km and *V*_max_) as well as the catalytic efficiencies, calculated as *V*_max_/Km, are presented in Table 1.

**Table 1:** Kinetic parameter values (Km, *V*_max_) and catalytic efficiencies (*V*_max_/Km) for in vitro conversion of profenofos to BCP

	Human	Rat
Liver microsomes		
Km (μM)	6.9	8.1
*V*_max_ (nmol/min/mg microsomal protein)	1.2	8.9
Catalytic efficiency (ml/min/mg protein)	0.17	1.1
Liver cytosol		
Km (μM)	1.4	0.34
*V*_max_ (nmol/min/mg cytosolic protein)	1.1	0.95
Catalytic efficiency (ml/min/mg protein)	0.79	2.8
Plasma		
Km (μM)	158	3.6
*V*_max_ (nmol/min/mg plasma protein)	0.99	0.68
Catalytic efficiency (ml/min/mg protein)	0.0063	0.19

The results indicate differences in the catalytic efficiencies for conversion of profenofos to BCP by rats and humans (Table 1). The catalytic efficiency for microsomal biotransformation of profenofos to BCP was 6.5-fold lower in incubations with human liver microsomes than with rat liver microsomes, due to a 7.7-fold lower Vmax and a 1.2-fold lower Km (Table 1). Likewise, the catalytic efficiency for cytosolic biotransformation of profenofos to BCP was 3.8-fold lower for human liver cytosol, with a 1.1-fold higher *V*_max_, and a 4.1-fold higher Km for human liver cytosol as compared to rat liver cytosol. Largest differences were observed for plasma, indicated by the 32-fold lower catalytic efficiency of biotransformation of profenofos to BCP for human plasma than for rat plasma, due to a 1.5-fold higher *V*_max_ and 44-fold higher Km for human plasma as compared to rat plasma.

### PBK model development and evaluation

**Fig. 4:**
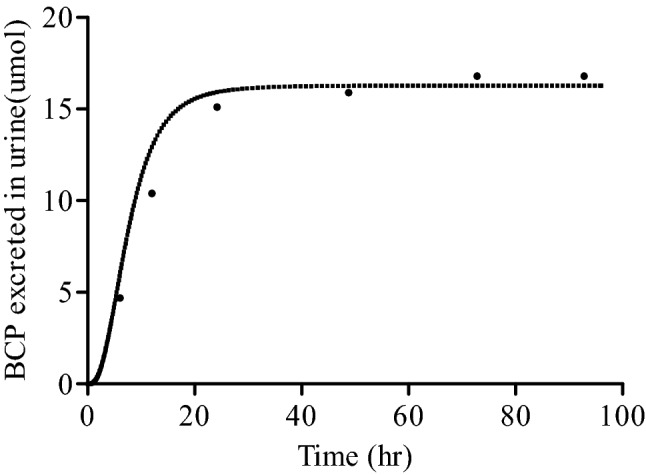
Comparison of PBK model-predicted (continuous line) and experimentally determined (symbols, Cho et al. 2002) time-dependent cumulative urinary excretion of BCP-glucuronide in rats upon oral administration of 35.8 mg profenofos/kg bw

Using the kinetic parameters defined in vitro and the input parameters obtained with in silico methods summarized in Supplementary Table 1, PBK models for profenofos in rat and human were made. Figure 4 presents the rat PBK model-based prediction of the urinary BCP excretion. In a subsequent step, first, the developed rat profenofos PBK model was evaluated against in vivo data on urinary excretion of conjugated BCP in rats from a study by Cho et al. (2002). These results indicate that the predictions made by the newly developed PBK model for rat match the reported in vivo data well. Due to a lack of in vivo kinetic data for humans, evaluation of the performance of the PBK model for profenofos in humans could not be performed. However, the human model was considered to be adequate, since it is based on the same conceptual model and in vitro- and in silico-derived input parameters were defined in the same way as those for the rat model.

**Fig. 5:**
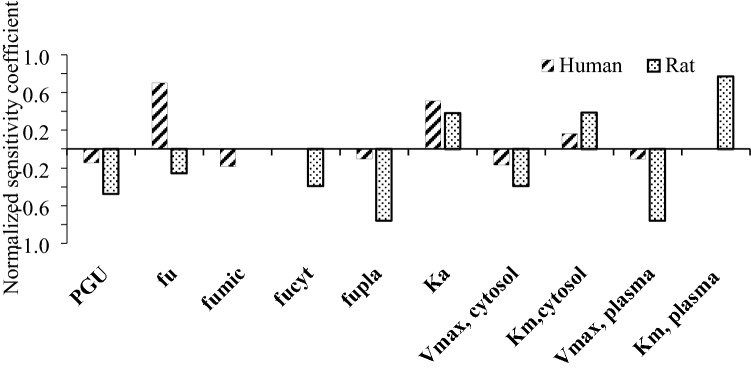
Sensitivity analysis representing the influence of model parameters on the predicted blood *C*_max_ of profenofos in humans and rats at predicted BMDL_10_ values of 0.01 and 0.45 mg/kg bw, respectively. *PGU *gut:plasma partition coefficient, *fu *fraction unbound in plasma, *fumic *fraction unbound in microsomal incubation, *fucyt *fraction unbound in cytosolic incubation, *fupla *fraction unbound in incubation with plasma, *ka *absorption constant, *V*_max_, cytosol = maximum rate of conversion of profenofos to BCP by liver cytosol, *Km* cytosol Michaelis–Menten constant for conversion of profenofos to BCP by liver cytosol, *V*_*max*_ plasma = maximum rate of conversion of profenofos to BCP by plasma, *Km* plasma = Michaelis–Menten constant for conversion of profenofos to BCP by plasma

Further evaluation of the PBK models included a local sensitivity analysis in which the impact of each chemical-specific parameter on the predicted free (unbound) *C*_max_ of profenofos in the rat and human PBK model was determined upon exposure to predicted effect doses (BMDL_10_ values derived from predicted dose–response data). Figure 5 presents the parameters for which the SCs are higher than 0.1 (absolute value). For both rat and human models, the prediction of the *C*_max_ of profenofos appeared to be mainly affected by the kinetic parameters for conversion of profenofos to BCP by enzymes from especially plasma and liver cytosol, in addition to the absorption rate constant (ka) and the gut to plasma partition coefficient.

**Fig. 6:**
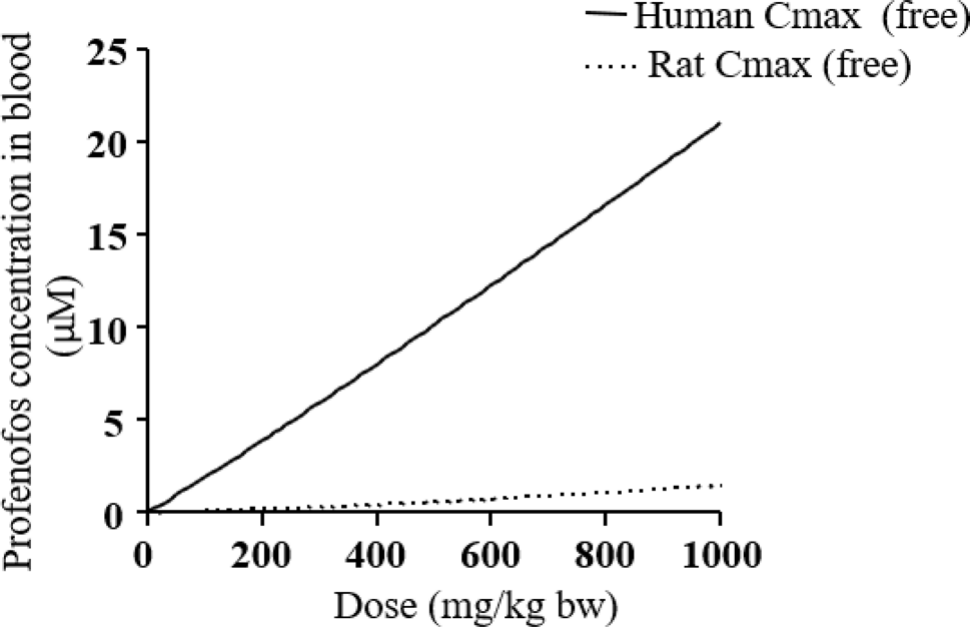
PBK model-predicted dose-dependent *C*_max_ (free) in blood upon oral profenofos exposure in rats (dotted line) and humans (continuous line)

The obtained PBK models reveal differences in kinetics in rats and humans (Fig. 6). Figure 6 shows the PBK model-predicted dose-dependent *C*_max_ values (free concentration) in rats and humans, indicating that humans are expected to reach around 20-fold higher blood concentrations than rats at equal oral exposure levels, mainly due to the lower catalytic efficiency for metabolic clearance of profenofos in humans.

### Prediction of in vivo dose-dependent AChE inhibition by PBK modeling-facilitated reverse dosimetry of in vitro data and comparison with reported in vivo effect data

**Fig. 7:**
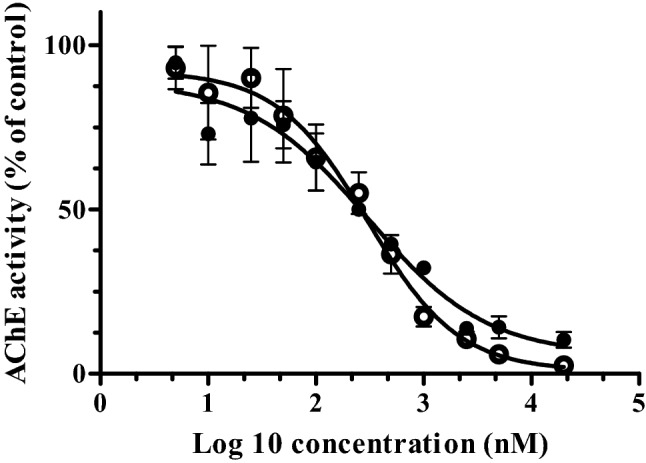
Acetylcholinesterase (AChE) activity in RBCs of Sprague–Dawley rats (closed symbols) and of human recombinant AChE (open symbols) with increasing concentrations of profenofos. AChE activity in the solvent control is set at 100%. Results represent data from 3 independent experiments and are presented as mean ± SEM

Concentration-dependent inhibition of rat RBC AChE (obtained from rat blood) and human (recombinant) AChE by profenofos were obtained in vitro (Fig. 7). Profenofos inhibited human AChE with an IC50 value of 302 nM, which is very close to the IC50 value of 350 nM reported by Das and Jamil (2006) using freshly obtained human erythrocytes. Profenofos inhibited rat AChE with an IC50 of 312 nM, suggesting no species difference in profenofos-induced AChE inhibition, based on effect concentrations related to 50% AChE inhibition.

**Fig. 8:**
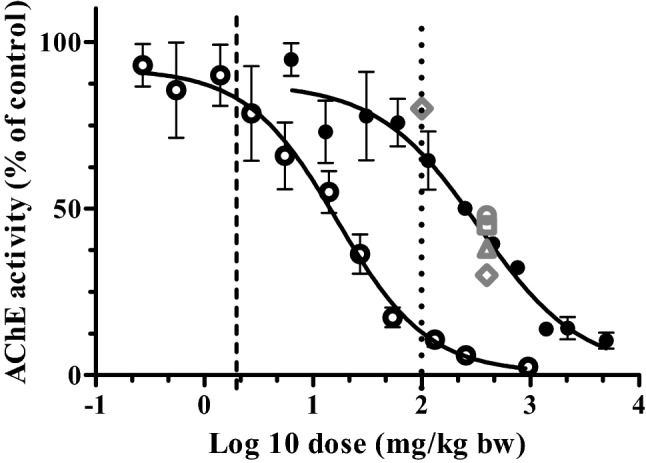
Predicted dose–response curves for profenofos-induced decrease of AChE activity in rats (closed black circles) and humans (open black circles). In vivo data on profenofos-induced AChE inhibition in rats as reported in JMPR (2007) are included: open grey diamond—male rat RBC inhibition, open grey triangle—male rat brain inhibition, open grey square—female rat brain inhibition, open grey circle—female rat RBC inhibition. EPA BMDL_10_ and JMPR NOAEL are indicated by the vertical lines (EPA 2016: dashed line; JMPR 2007: dotted line)

The concentration-dependent AChE inhibition curves obtained in this study were converted into in vivo dose-dependent AChE inhibition curves in rats and humans (Fig. 8) using the PBK modeling-facilitated reverse dosimetry approach as described in the materials and methods section. Figure 8 also presents in vivo data for male and female rat profenofos-induced AChE inhibition in RBCs and in the brain available from literature (JMPR 2007). The results reveal that predicted in vivo dose–response curves for rat AChE inhibition are close to the reported in vivo data for male and female rat AChE inhibition in RBCs and in the brain (JMPR 2007), indicating that with this combined in vitro–in silico approach, good predictions for rats were obtained. Predictions for humans indicate that humans are expected to be more sensitive than rats regarding profenofos-induced AChE inhibition (Fig. 8), which is mainly due to the slower metabolic clearance of profenofos, resulting in higher *C*_max_ values at the same dose levels (Fig. 6). Toxicity data for humans for further evaluation of the predicted dose–response curves are not available.

BMD analysis was performed on predicted dose–response data for rats and human. Results of these analyses are presented in Supplementary Tables 2 and 3 and Supplementary Figs. 2 and 3. BMDL_10_ values obtained for rats and humans upon model averaging were 0.45 and 0.01 mg/kg bw, respectively, predicting humans to be more sensitive than rats. In theory, BMD values obtained from predicted dose–response data may serve as PODs for setting safe exposure levels, i.e., an ARfD, and these BMD values were, therefore, compared with PODs that have been used by the US EPA, JMPR and EFSA in their assessments.

The US EPA considered erythrocyte (RBC) AChE inhibition to be more suitable to derive a POD for human safety assessments, since it is more sensitive than brain AChE inhibition in case of profenofos exposure (EPA 1999, 2016). A 10% RBC inhibition of AChE has been adopted by the US EPA to obtain a POD to define the ARfD for profenofos (EPA 2016). The US EPA reported a BMDL_10_ of 1.99 mg/kg bw for profenofos-induced RBC AChE inhibition in adult rats upon a single exposure to profenofos, being 4 times higher than the BMDL_10_ obtained from the predicted dose–response data for rats in our study (0.45 mg/kg bw).

JMPR used a NOAEL value of 100 mg/kg bw to derive an ARfD, based on a dataset on profenofos-induced inhibition of brain AChE in rats (upon a single exposure to profenofos). This NOAEL value is 50-fold higher than the BMDL_10_ value reported by the US EPA and 200-fold higher than the BMDL_10_ value obtained from our predicted dose–response data in rats (0.45 mg/kg bw), suggesting the NOAEL to result in a POD that may be too high.

In a recent report from EFSA (EFSA 2019), an ARfD for profenofos of 0.005 mg/kg bw was reported, based on a German evaluation in 2001 that defined an ARfD based on a NOAEL in a dog study using inhibition of brain cholinesterase activity as critical effect. Unfortunately, no further information (e.g., NOAEL) on that particular dog study is publicly available. If a safety factor of 100 was used to derive this ARfD, the NOAEL derived from the dog study would be close to the BMDL_10_ value obtained from our predicted dose–response data in rats (0.45 mg/kg bw (~ 100-fold higher than the ARfD)).

## Discussion

The aim of the current study was to develop a physiologically based kinetic (PBK) model in rats and humans solely based on in vitro and in silico input parameters to be applied for the prediction of in vivo AChE inhibition by the OP pesticide profenofos by reverse dosimetry of in vitro AChE inhibition data. To this end, profenofos PBK models were developed for rats and humans and used to predict dose-dependent internal profenofos concentrations, which were applied for reverse dosimetry to translate in vitro concentration-dependent data on profenofos-induced inhibition of AChE to in vivo dose–response curves for profenofos-induced inhibition of AChE in blood. The results indicate that profenofos-induced AChE inhibition in rats was closely predicted and that humans are predicted to be more sensitive to profenofos-induced AChE inhibition than rats upon acute exposure.

This study has shown marked interspecies differences in toxicokinetics of profenofos with predicted blood *C*_max_ values for profenofos in humans being around 20-fold higher than in rats at equal levels of exposure (Fig. 6). This is mainly due to higher catalytic efficiencies for microsomal, cytosolic and plasma biotransformation of profenofos to BCP in rats as compared to humans as observed in this study (Table 1). Profenofos, being an oxon, is mainly detoxified through hydrolysis by hepatic and plasma PON1 and CYP450-mediated detoxification, resulting in the production of BCP (JMPR 2007; EPA 2016; Dadson et al. 2013). Interspecies differences in activities between human and rat PON1 have been reported, with human PON1 having lower catalytic activity as compared to rat PON1 (Kaliste-Korhonen et al. 1996). Another possible reason for the differences in profenofos detoxification may be related to quantitative differences in B-esterases in rats and humans, which may affect the metabolism and disposition of ester compounds including OPs (Ecobichon and Comeau 1973; Maxwell et al. 1987). B-esterases, such as carboxylesterase (CaE), butyrylcholinesterase (BuChE) and acetylcholinesterase (AChE), detoxify oxons, but these enzymes are inhibited by the oxons as a consequence (Chanda et al. 1997). It has been reported that rat plasma contains almost all types of esterases including CaE, PON1, BChE, and AChE (Bahar et al. 2012; Satoh and Hosokawa 2006), whereas humans are deficient of plasma CaE (Williams et al. 2011; Berry et al. 2009; Li et al. 2005) and plasma AChE (Ecobichon and Comeau 1973). The relevance of CaE in OP detoxification is indicated by a study that demonstrated that in vivo inhibition of CaEs by cresylbenzodioxaphosphorin oxide (CBDP) caused an increase in the toxicity of many OPs in rats, mice, rabbits and guinea pigs (Ecobichon and Comeau 1973; Maxwell et al. 1987). In the same study, the interspecies differences observed in the toxicity of OPs were lost in the group treated with the in vivo CaE inhibitor (CBDP) (Maxwell et al. 1987). The absence of CaE in human plasma in addition to low PON1 activity may, therefore, be responsible for lower profenofos clearance resulting in higher sensitivity of humans to profenofos as compared to rodents (Kaliste-Korhonen et al. 1996). In this way, B-esterases may play a protective role in rats but not humans.

Data on RBC AChE inhibition can be considered as an appropriate surrogate measure of potential organophosphate effects on the peripheral and central nervous systems in absence of brain AChE inhibition data (Chen et al. 1999; Das and Jamil 2006). To predict profenofos dose levels that result in AChE inhibition in rats and humans, profenofos in vitro concentration–response curves for RBC AChE inhibition (Fig. 7) were translated to in vivo dose–response curves for RBC AChE inhibition for both rats and humans (Fig. 8). The predicted data thus obtained for the rat matched with the available in vivo rat data as reported by JMPR (2007) (Fig. 8), and the BMDL_10_ obtained from the predicted dose–response data differed only fourfold from the BMDL_10_ obtained from data from an in vivo rat study on profenofos-induced blood AChE inhibition used by the US EPA for determination of an ARfD for profenofos. This indicates that in vivo dose-dependent AChE inhibition was closely predicted based on our approach, giving confidence in the human predictions, which could not be further evaluated because of the lack of human in vivo data on this endpoint. Our predictions suggest that humans are more sensitive than rats towards profenofos-induced AChE inhibition. The interspecies differences (45-fold difference in BMDL_10_) were predicted to be mainly due to interspecies differences in toxicokinetics, as discussed above, and these would not be covered by the standard uncertainty factor of 10 to account for interspecies differences. Currently, we work on the assessment of human interindividual differences in profenofos metabolic clearance (using plasma and liver fractions of different donors) to be incorporated in PBK models, providing insight into whether a combined uncertainty factor of 100 (10 for interspecies differences and 10 for intraspecies differences) applied to the rat AChE inhibition data is expected to be sufficiently protective for sensitive human individuals.

In the present study, the BMDL_10_ calculated based on predicted rat data was 4 times lower than the BMDL_10_ value used by EPA and 200-fold lower than the NOAEL used by JMPR to obtain an ARfD, implying that our prediction is more conservative as compared to the rat data used by the two agencies. In a possible future risk assessment paradigm that would be independent of animal data, BMDL_10_ values obtained from the predicted human dose–response data may be applied. In the present study, we determined a BMDL_10_ value for ‘an average human’ (0.01 mg/kg bw), not quantifying possible interindividual differences. To take interindividual differences into account, an uncertainty factor may be applied, but it would be scientifically more sound to quantify interindividual differences in the human population in profenofos detoxification. This is especially of interest given the reported genetic polymorphisms of human PON1 (and BuChE) with related phenotypes of low and high activities (Schwarz et al. 1995; Geldmacher et al. 1989), which may play a role in the interindividual differences in PON1 activity (Darney et al. 2020), suggesting possible large human interindividual differences in profenofos detoxification. As indicated above, we currently work on the determination of such interindividual differences in profenofos detoxification in humans and integrate these data in the human PBK modeling-based predictions of profenofos toxicity.

Although the current models quantitatively predicted profenofos tissue dosimetry and the resulting AChE inhibition, possible limitations of the approach should be considered. In the current PBK model, gut absorption of profenofos was estimated to be 100% using a first-order process with an absorption rate constant of 1 h^−1^. Although this resulted in the adequate prediction of time-dependent BCP-glucuronide in rat urine, it must be noted that that specific model output is not so sensitive to the ka (data not shown), whereas the (unbound) *C*_max_ is (Fig. 5). It is noteworthy that the extent of absorption varies depending on the dosing method, dose formulation (solution) and variations in species, strain and gender (Kararli 1995). Furthermore, profenofos absorption as present in the food matrix may be different.

We used a static approach to determine the AChE inhibitory effect concentrations, using a single pre-incubation time point (15 min). When including more time points, information on the time-dependent inhibition kinetics can be obtained. However, a previous study with chlorpyrifos oxon showed that human recombinant AChE active sites were 100% inhibited after a pre-incubation period of 11 min (Kaushik et al. 2007). One would expect that effect concentrations would decrease with longer incubation times until 100% of the binding sites is inhibited, as shown before by Aurbek et al. (2006) and Krstić et al. (2008). With a relatively long pre-incubation time of 15 min as we have used in the present study, similar to incubation times previously applied by other research groups (e.g., Aurbek et al. 2006; Kasteel et al. 2020), we aimed to obtain a relevant and conservative estimation of the effect concentrations, as also indicated by the small difference between the BMDL_10_ value obtained with our predicted rat dose–response data and the BMDL_10_ value from a reported in vivo study in rats.

It must also be noted that the approach used in this study may be suitable to estimate AChE inhibition upon a single exposure, while not predicting effects upon repeated exposure, since upon longer exposures, one must take the time-dependent de novo AChE production into account to adequately estimate the AChE activity upon a second and further exposure. In that regard, a phenomenon referred to as steady state AChE inhibition is of interest, which is the situation when the degree of AChE inhibition reaches equilibrium with the production of new enzyme at which AChE inhibition remains constant at a specified dose over the exposure period (EPA 2016). Adequate prediction of these processes based on only in vitro data, as applied in the present study, seems at this stage not possible. Furthermore, one must consider that OPs inhibit detoxifying enzymes, such as CaE and BuChe, which may result in a decrease in metabolic clearance, and related higher internal concentrations and increased sensitivity upon repeated dosing.

Another limitation of the approach is that the prediction of OP toxicity was only based on AChE inhibition as the endpoint. It should, however, be noted that OPs may affect a number of additional targets (such as Neuropathy target esterase (NTE) (Costa 2018), muscarinic M2 receptors (Costa 2006), acylpeptide hydrolase (APH) (Richards et al. 2000), fatty acid amide hydrolase (FAAH) (Quistad et al. 2001; Buntyn et al. 2017) and a variety of lipases (Quistad et al. 2006)) that lead to OP toxicity, including neuroinflammation, autoimmunity and axonal transport deficits (Naughton and Terry 2018). On the other hand, AChE inhibition has been used as an endpoint to set a POD for the risk assessment (EPA 2016; EFSA 2019), indicating its relevance. The QIVIVE approach could be extended in the future to also predict dose-dependent effects related to other OP targets, allowing a more extensive hazard assessment based on NAMs.

In conclusion, the predicted dose-dependent profenofos-induced AChE inhibition in rats are close to reported data on dose-dependent in vivo AChE inhibition in rats upon single dosing, providing also confidence in the predictions obtained for humans. Results from this study suggest that humans may be more sensitive to AChE inhibition upon profenofos exposure than rats, which is mainly due to differences in profenofos detoxification. Altogether, the results demonstrate the ability to predict in vivo AChE inhibition by profenofos, providing another proof-of-principle that with NAMs in vivo effects of chemicals can be predicted without the use of in vivo studies.

## Supplementary Information

Below is the link to the electronic supplementary material.

Supplementary file1 (DOCX 480 KB)
