# Supplementary material for: Prediction of dose-dependent in vivo acetylcholinesterase inhibition by profenofos in rats and humans using physiologically based kinetic (PBK) modeling-facilitated reverse dosimetry
Source: Arch Toxicol. 2021 Mar 2;95(4):1287–301. doi: 10.1007/s00204-021-03004-4 (PMC8032624; doi:10.1007/s00204-021-03004-4)
Supplement: Supplementary file 1 — Supplementary file1 (DOCX 480 KB) [file 204_2021_3004_MOESM1_ESM.docx]

**Prediction of dose-dependent in vivo acetylcholinesterase inhibition by profenofos in rats and humans using physiologically based kinetic (PBK) modeling-facilitated reverse dosimetry**

Isaac Omwenga^a,b*^, Shensheng Zhao^a^, Laetitia Kanja^b^, Hans Mol^c^, Ivonne M.C.M. Rietjens^a^, Jochem Louisse^c^

^a^ Division of Toxicology, Wageningen University and Research, Stippeneng 4, 6708 WE, Wageningen, The Netherlands

^b^ Department of Public Health, Pharmacology and Toxicology, Faculty of Veterinary Sciences, University of Nairobi, P.O. Box 29053-00625, Nairobi, Kenya

^c^ Wageningen Food Safety Research, Wageningen University and Research, Akkermaalsbos 2, 6708 WB, Wageningen, The Netherlands

^d^ Department of Animal Science, Meru University of Science and Technology, P.O Box, 972-60200 Meru, Kenya.

*****Corresponding author

Isaac Omwenga

Division of Toxicology, Wageningen University and Research

PO BOX 8000, 6708 EA Wageningen,

The Netherlands

E-mail address: isaac.omwenga@wur.nl

Supplementary Table 1. Physiological and chemical-specific parameters used for the PBK models.

| **Model parameter** | **Human** | **Rat** |
| --- | --- | --- |
| **Physiological parameters** | | |
| Body weight  Fractional tissue volumes | 70 | 0.20 |
| Fat | 0.213 | 0.07 |
| Bone | 0.0856 | 0.0659 |
| Brain | 0.02 | 0.0052 |
| Gut | 0.0171 | 0.026 |
| Heart | 0.0057 | 0.0044 |
| Kidney | 0.0044 | 0.0092 |
| Liver | 0.021 | 0.036 |
| Lung | 0.0076 | 0.0052 |
| Muscle | 0.4 | 0.488 |
| Skin | 0.0371 | 0.1656 |
| Spleen | 0.0026 | 0.0024 |
| Venous blood | 0.0514 | 0.0429 |
| Arterial blood | 0.0257 | 0.0215 |
| Rest of body | 0.1098 | 0.0577 |
|  |  |  |
| Cardiac output (mL/s) | 108.33 | 1.33 |
| Fractional tissue blood flows |  |  |
| Fat | 0.05 | 0.059 |
| Bone | 0.05 | 0.10 |
| Brain | 0.12 | 0.014 |
| Gut | 0.1465 | 0.0101 |
| Heart | 0.04 | 0.04 |
| Kidney | 0.19 | 0.145 |
| Liver (Venous side) | 0.2154 | 0.242 |
| Lung | 1 | 1 |
| Muscle | 0.17 | 0.237 |
| Skin | 0.05 | 0.051 |
| Spleen | 0.0172 | 0.011 |
| Rest of body | 0.02119 | 0.112 |
| Renal clearance (L/hr) | 6.7 | 0.078 |
| **Chemical specific parameters profenofos** | | |
| Molecular weight | 373.63 | |
| LogP | 4.88 | |
| pKa | NA | |
| LogD*(olive oil: water) | 4.09 | |
| Binding data |  |  |
| Fu blood | 0.034 | |
| Fraction unbound microsomes | 0.07^a^ | 0.232^b^ |
| Fraction unbound cytosol | 0.07^c^ | 0.232^d^ |
| Fraction unbound plasma (in vitro incubations) | 0.83^e^ | 0.93^f^ |
| Tissue to plasma partition coefficients | | |
| Fat | 96.3 | 147 |
| Bone | 15.4 | 11.9 |
| Brain | 14.1 | 23.3 |
| Gut | 11.1 | 14.1 |
| Heart | 3.47 | 7.47 |
| Kidney | 5.35 | 8.84 |
| Liver | 8.82 | 9.70 |
| Lung | 1.24 | 11.1 |
| Muscle | 5.43 | 5.44 |
| Skin | 6.62 | 12.4 |
| Spleen | 5.45 | 5.05 |
| Rest of body | 1 | 1 |
| Absorption Ka (/hr) | 1 | 1 |
| Fraction absorbed | 1 | 1 |
| **Chemical specific parameters: BCP** | | |
| LogP | - | 3.04 |
| pKa | - | 7.54 |
| LogD*(olive oil: water) | - | 1.80 |
| Tissue plasma partition coefficients | | |
| Fat | - | 5.62 |
| Bone | - | 2.90 |
| Brain | - | 5.65 |
| Gut | - | 3.54 |
| Heart | - | 2.05 |
| Kidney | - | 2.36 |
| Liver | - | 2.52 |
| Lung | - | 2.87 |
| Muscle | - | 1.58 |
| Skin | - | 3.11 |
| Spleen | - | 1.49 |
| Rest of body | - | 1 |
| Binding data | | |
| Fu blood | - | 0.113 |
| Fraction unbound microsomes | - | 0.761^g^ |
| **Chemical specific parameters: BCP glucuronide** | | |
| LogP | - | 1.06 |
| pKa | - | 2.72 |
| LogD* (olive oil: water) | - | 4.85 |
| Tissue to plasma partition coefficients | | |
| Fat | - | 0.13 |
| Bone | - | 0.79 |
| Brain | - | 1.49 |
| Gut | - | 1.16 |
| Heart | - | 1.00 |
| Kidney | - | 1.05 |
| Liver | - | 1.01 |
| Lung | - | 1.12 |
| Muscle | - | 0.93 |
| Skin | - | 1.05 |
| Spleen | - | 1.02 |
| Rest of body | - | 1 |
| Binding data | | |
| Fu blood | - | 0.226 |
| **Scaling factors** |  |  |
| Microsomal protein (mg) per g liver | 32 | 35 |
| Cytosolic protein (mg) per g liver | 80.7 | 80.7 |
| Plasma (mg)per g blood | 550 | 550 |

^a^ fraction unbound profenofos in human microsome incubation (with 2 mg/mL)

^b^ fraction unbound profenofos in rat microsome incubation (with 0.5 mg/mL)

^c^ fraction unbound profenofos in human cytosol incubation (with 2 mg/mL)

^d^ fraction unbound profenofos in rat cytosol incubation (with 0.5 mg/mL)

^e^ fraction unbound profenofos in human plasma incubation (estimated with help of simcyp (via Kd: 13 µM); 4.4 mg plasma/mL (0.20 g albumin/L))

^f^ fraction unbound profenofos in rat plasma incubation (estimated with help of simcyp (via Kd: 13 µM); 1.65 mg plasma/mL (0.074 g albumin/L))

^g^ fraction unbound BCP in rat microsome incubation (with 1 mg/mL)

Lung

Rest of body

Fat

Bone

Brain

Heart

Kidney

Muscle

Skin

Spleen

Liver

Lung

Fat

Bone

Brain

Heart

Kidney

Muscle

Skin

Spleen

Liver

Lung

Rest of body

Fat

Bone

Brain

Heart

Kidney

Muscle

Skin

Spleen

Liver

BCP

BCP

Glucuronide

Oral Dose

GI tract

BCP glucuronide in Urine

GI tract

GI tract

Supplementary Figure 1. Schematic diagram of the PBK model for profenofos for rat consisting of three sub-models including one for profenofos (left), 4-bromo-2-chlorophenol (BCP) (center) and for BCP glucuronide (right). BCP formation is also described in the blood compartments in the PBK model, but for the sake of clarity of the figure, this is not depicted in the schematic diagram.

Abbreviation: GI tract: gastrointestinal tract.

Supplementary Table 2. Results of BMD modelling of predicted human dose-response data, applying model averaging

Fitted Models

| model | converged | loglik | npar | AIC |
| --- | --- | --- | --- | --- |
| full model | Yes | 17.79 | 13 | -9.58 |
| null model | Yes | -57.88 | 2 | 119.76 |
| Expon. m3- | Yes | -1.13 | 4 | 10.26 |
| Expon. m5- | Yes | 12.71 | 5 | -15.42 |
| Hill m3- | Yes | -0.61 | 4 | 9.22 |
| Hill m5- | Yes | 13.53 | 5 | -17.06 |
| Inv.Expon. m3- | Yes | 4.78 | 4 | -1.56 |
| Inv.Expon. m5- | Yes | 13.13 | 5 | -16.26 |
| LN m3- | Yes | 2.16 | 4 | 3.68 |
| LN m5- | Yes | 13.47 | 5 | -16.94 |
| Weights for Model Averaging | | | | |
| EXP | HILL | INVEXP | LOGN | EXP |
| 0.14 | 0.33 | 0.22 | 0.31 | 0.14 |
| Final BMD Values | | | | |
| endpoint | subgroup | BMDL | BMDU |  |
| Response | All | 0.01 | 0.81 |  |


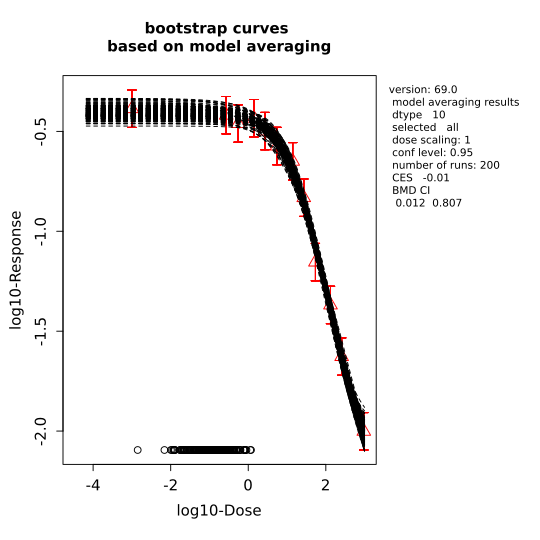


Supplementary Figure 2. Bootstrap curves based on model averaging for predicted human AChE inhibition.

A B


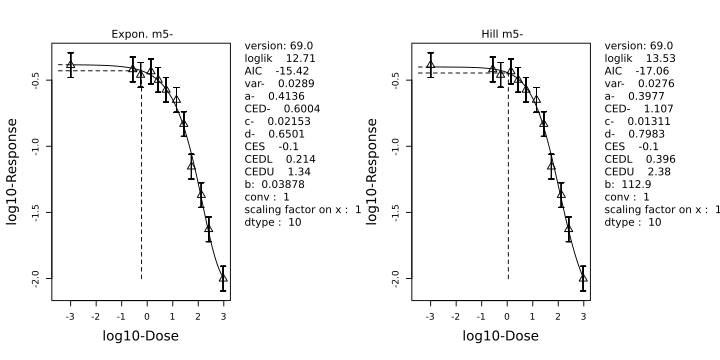


C D


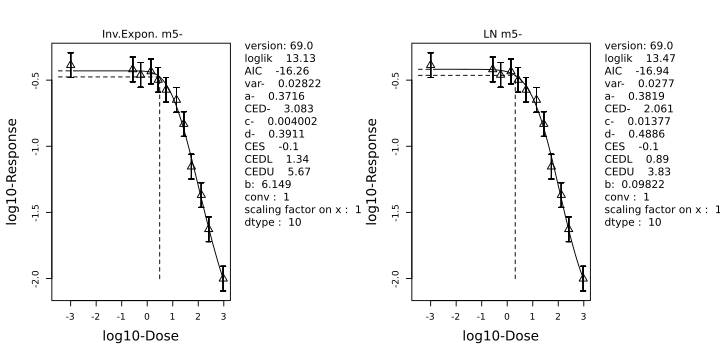


Supplementary Figure 3. BMD modelling results for individual models for predicted human AChE inhibition. A: Exponential model 5, B: Hill model 5, C:Inv. Exponential model 5, D: Log-Normal Family model 5.

Supplementary Table 3. Results of BMD modelling of predicted rat dose-response data, applying model averaging.

### Fitted Models

| model | converged | loglik | npar | AIC |
| --- | --- | --- | --- | --- |
| full model | yes | -10.87 | 13 | 47.74 |
| null model | yes | -44.71 | 2 | 93.42 |
| Expon. m3- | yes | -15.31 | 4 | 38.62 |
| Expon. m5- | yes | -12.87 | 5 | 35.74 |
| Hill m3- | yes | -15.25 | 4 | 38.50 |
| Hill m5- | yes | -13.15 | 5 | 36.30 |
| Inv.Expon. m3- | yes | -14.61 | 4 | 37.22 |
| Inv.Expon. m5- | yes | -13.70 | 5 | 37.40 |
| LN m3- | yes | -14.87 | 4 | 37.74 |
| LN m5- | yes | -13.38 | 5 | 36.76 |
| Weights for Model Averaging | | | | |
| EXP | HILL | INVEXP | LOGN | EXP |
| 0.35 | 0.27 | 0.17 | 0.21 | 0.35 |
| Final BMD Values | | | | |
| endpoint | subgroup | BMDL | BMDU |  |
| Response | all | 0.45 | 190 |  |


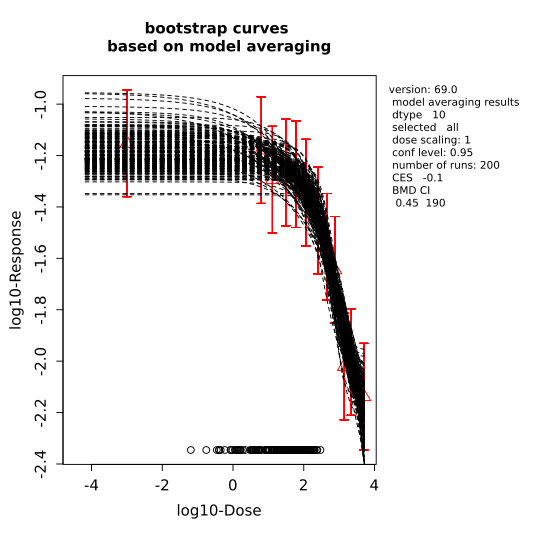


Supplementary Figure 4. Bootstrap curves based on model averaging for predicted rat AChE inhibition.

A B


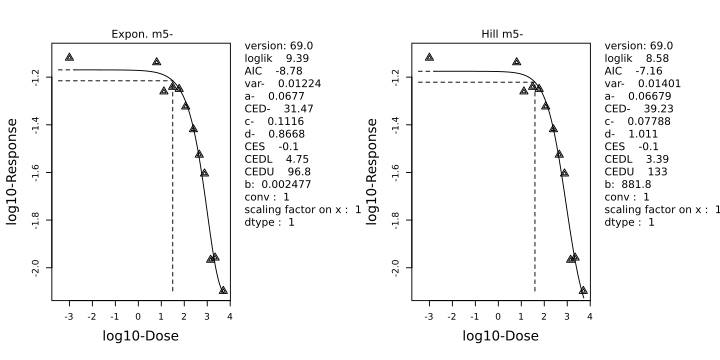


C D


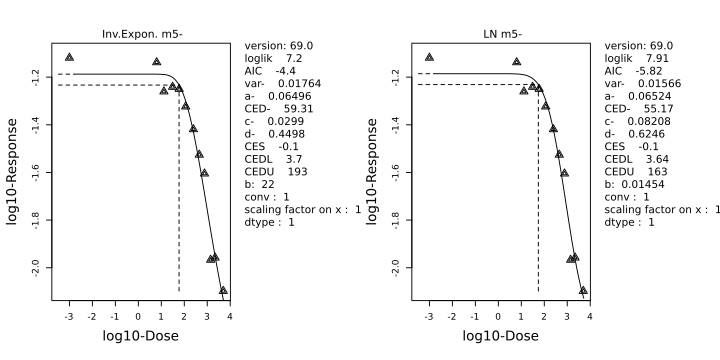


Supplementary Figure 5. BMD modelling results for individual models for predicted rat AChE inhibition. A: Exponential model 5, B: Hill model 5, C: Inv. Expontial model 5, D: Log-Normal Family model 5.
